# Im Osten was Neues – Agile Transformation bei KOMSA

**DOI:** 10.1007/s11613-021-00746-z

**Published:** 2022-01-08

**Authors:** Pierre-Pascal Urbon

**Affiliations:** Weidlingstraße 4, 34119 Kassel, Deutschland

**Keywords:** Agilität, Leadership, Transformation, Change, ITK, Agiliy, Leadership, Transformation, Change, ICT

## Abstract

Der europäische ITK-Dienstleister KOMSA setzte unter Führung des neuen Vorstandsvorsitzenden und Finanzvorstands Pierre-Pascal Urbon mit dem Projekt Transformation 2020 in nur 7 Monaten die größte Veränderung seit der Unternehmensgründung um. Mit den Ideen und Impulsen der Mitarbeiter hat KOMSA die Komplexität reduziert und eine neue Führungs- und Steuerungsfunktion eingeführt. Im Rahmen des Projekts führte der Vorstand eine Leadership Journey durch, um ein neues Führungsverständnis zu vermitteln. Die Umsetzung erfolgte mit agilen Methoden, sowohl vor Ort als auch aus dem Home-Office. Der Artikel beschreibt die Ausgangslage, die Mentalität der Mitarbeiter des sächsischen Familienunternehmens, den Prozess sowie die Sichtweisen der Aufsichtsratsvorsitzenden Kerstin Grosse und der Führungskraft Andrea Fiedler-Braunschweig.

## Abstract (en)

KOMSA, a European leading ICT service provider, completed with the project Transformation 2020 the biggest change process in the company’s history within only 7 months. The new CEO and CFO Pierre-Pascal Urbon capitalized the ideas and impulses of employees to reducing the complexity of the group and to implementing a new leadership and management system. During the project the management board conducted the Leadership Journey to convey the new leadership mindset. The project has been implemented with agile methods on site as well as remote in home offices. This article describes the base line, mindset of the employees of the family-owned business in Saxony, Germany, and the process as well as the views of Kerstin Grosse, chairwoman of the supervisory board, and of Andrea Fiedler-Braunschweig, Vice President of the Company.

## KOMSA-Gründung

**Figure 1:**
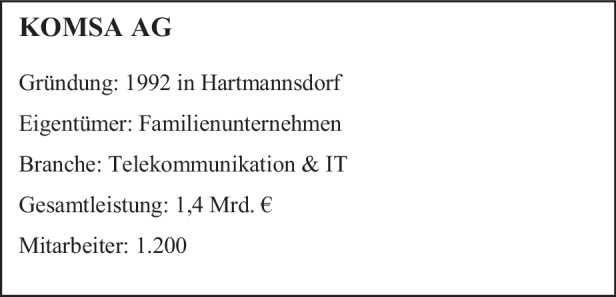

Die Jahre nach der Wiedervereinigung waren für die neuen Bundesländer schwierig. Die Zahl der Angestellten in den volkseigenen Betrieben (VEB) wurde nach der Wende von 4,1 Mio. um über 70 % abgebaut. Rund die Hälfte der etwa 8500 VEB wurden geschlossen. 1992 lag die Arbeitslosigkeit in den neuen Bundesländern bei 14,4 %. In dieser Zeit des Umbruchs gründete der gebürtige Schwede Gunnar Grosse mit drei Mitstreitern auf einem Bauernhof im sächsischen Hartmannsdorf die KOMSA. Das Gründerteam hatte die Vision, die damals noch junge Mobiltelefonie nach Deutschland zu bringen. KOMSA war einer der ersten Distributoren, der sich am Aufbau eines Mobilfunk-Händlernetzes beteiligte. Heute umfasst das Portfolio mehr als 25.000 Artikel von über 200 Informations- und Telekommunikations (ITK)-Herstellern. Mit Hard- und Software bedient das Unternehmen über 20.000 Handelspartner und ca. 2000 Fachhändler. Die mehrfach ausgezeichnete Logistik versendet europaweit täglich bis zu 25.000 Pakete an Endverbraucher. Die Marken KOMSA und aetka zählen zu den Branchenbesten (Abb. 1).

Das Gründerteam hat die an die ITK-Distribution angrenzenden Geschäftsfelder im Laufe der Jahre konsequent erschlossen. So bietet KOMSA heute z. B. auch die Reparatur von elektronischen Geräten, Professional Managed Services rund um mobile Geräte sowie eine online-Plattform für den An- und Verkauf von aufbereiteten Smartphones und Tablets an.

## Durch Generationenwechsel neues Governance Modell

2017 setzte das Gründerteam den Generationenwechsel um. Die im Vorstand verbliebenen Gründer zogen sich in den Aufsichtsrat zurück. Das dreiköpfige Aufsichtsratsgremium wird von Kerstin Grosse geleitet. Der neue Vorstand bestand zunächst aus fünf Führungskräften der KOMSA, die schon viele Jahre im Unternehmen erfolgreich tätig waren.

Um eine neue Entwicklungsphase der KOMSA einzuleiten, beschloss der Aufsichtsrat im Herbst 2019 den Vorstand zu verkleinern und die Position des Vorstandsvorsitzenden neu zu besetzen. Zum 1. Mai 2020 wurde ich vom Aufsichtsrat als neuer Vorstandsvorsitzender (CEO) und Finanzvorstand (CFO) bestellt. Bereits in den Vertragsverhandlungen diskutierten der Aufsichtsrat und ich das Rollenverständnis und die Verantwortlichkeiten. Wir wollten zukünftig die Vorteile eines dualistischen Systems nutzen und die Durchführung der Geschäftsführung von der Kontrolle trennen. Um wirkungsvoll in den jeweiligen Rollen agieren zu können, ist ein strukturierter und regelmäßiger Informationsaustausch zwischen Vorstand und Aufsichtsrat wichtig. Durch das gemeinsam erarbeitete Verständnis zum neuen Governance Modell konnten wir die Strukturvorteile eines Familienunternehmens (z. B. die intensivere Einbeziehung der Gründer in die Unternehmensentwicklung sowie die Langfristperspektive) mit den sinnvollen Elementen börsennotierter Gesellschaften (z. B. die höhere Transparenz und strikte Trennung von Eigentum und Verfügungsmacht) verbinden.

## Neuausrichtung mitten in der Covid-Pandemie

Im April 2020 begann meine Einarbeitung. Als branchenfremder Manager musste ich mich nicht nur mit der unternehmensspezifischen Situation der KOMSA und mit den Vorstellungen der Mitarbeitenden, sondern auch mit den Branchentrends und der Industrielogik vertraut machen. Für eine präzise Praxisanalyse und ein besseres Verständnis der Verhaltensweisen führte ich im ersten Schritt über 60 halb-strukturierte und leitfadenorientierte Interviews. Der Leitfaden diente der Vorstrukturierung der Informationen und ermöglichte mir die Vergleichbarkeit der qualitativen Aussagen. Die Themenschwerpunkte teilte ich meinen Gesprächspartnern zur Vorbereitung mit. Neben den strukturierten Fragen ließ ich im Gespräch Freiraum für das unstrukturierte Erzählen zu einem Thema. Dadurch konnte ich die Reichweite und die Tiefe des Themas besser abdecken. Um eine entspannte Gesprächsatmosphäre herzustellen, legte ich Wert auf einen persönlichen Austausch und eine wertschätzende Haltung. Zu Beginn des Gesprächs mit Mitarbeitenden ging ich auf meine eigenen emotionalen Aspekte ein und schilderte mein Werteverständnis anhand von Beispielen. In den Interviews begegnete ich Menschen, die mit Offenheit und hoher Fachkompetenz über die aktuellen Chancen und Probleme der Branche und von KOMSA berichteten. Häufig entstand eine Vertrautheit, in der ich auch Verhaltensmuster und Einstellungen ansprechen konnte.

Zu den Interviewpartner zählten Vorstände, Führungskräfte, Mitarbeiter, Betriebsräte, Team-Coaches^1^ und Auszubildende sowie Hersteller, Netzbetreiber, Kunden, Wirtschaftsprüfer und Finanzierungspartner. In meiner Praxisanalyse führte ich die jeweiligen qualitativen Bildausschnitte eines jeden Interviews zu einem Gesamtbild zusammen: KOMSA war viel zu komplex organisiert und konnte daher auf Marktveränderungen nur unzureichend reagieren. Durch die Vielzahl der Konzerngesellschaften dauerte die interne Abstimmung teilweise mehrere Tage, während die Kunden die Entscheidungen in wenigen Stunden treffen konnten. Neueinstellungen waren in der Regel die Antwort auf neue Aufgabenstellungen. Die zahlreichen Überlappungen und internen Leistungsverrechnungen beeinträchtigten die Produktivität und die Transparenz. Die Probleme der Komplexität spürte nahezu jeder Mitarbeiter in der täglichen Arbeit. KOMSA erwirtschaftete aufgrund der gewachsenen Strukturen zu wenig Gewinn im Vergleich zum Wettbewerb. Die sich abzeichnenden Wachstumspotenziale aus der Konvergenz von IT und Telekommunikation sowie von Professional Managed Services und der Kreislaufwirtschaft konnte KOMSA in der gewachsenen Struktur nicht vollständig nutzen. Eine fundamentale Transformation der KOMSA war notwendig, um den disruptiven Veränderungen im Handel nachhaltig zu begegnen.

Der Zeitpunkt für eine Transformation war denkbar ungünstig. Die Covid-19 Pandemie beschleunigte den Umbruch im Handel. Durch den Lockdown brachen die Umsätze im stationären Handel ein, während sich Online-Marktplätze, Cloud-Anwendungen für dezentrales Arbeiten und Lernen in kürzester Zeit zum Standard entwickelten. Zudem war die Verunsicherung bei den Mitarbeitenden durch den Vorstandsumbau, Kurzarbeit und Home-Office groß. Kurz, alles das, was über viele Jahre vertraut war, war plötzlich unsicher und gefährdet.

Bei der Entscheidung über die weitere Vorgehensweise spielte die Unternehmenskultur eine große Rolle. Trotz der Unternehmensgröße hatte KOMSA viele Elemente einer Start-up Kultur beibehalten. Hierzu zählten beispielsweise die flache Hierarchie, Du-Kultur und offene Kommunikation. Umbrüche konnten auch in der Vergangenheit gut verarbeitet werden. Die Mitarbeitenden waren daher gegen transformationsbedingte Risiken weitgehend immunisiert, solange sie in den Prozess eingebunden waren und die Entscheidungen mitgestalten konnten.

**Figure 2:**
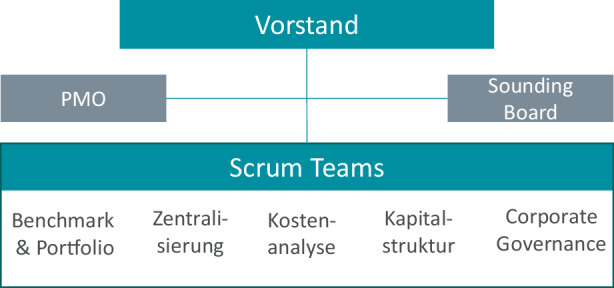

Auf Basis der Erkenntnisse meiner Praxisanalyse entschieden wir im Vorstand, bei der Umsetzung der notwendigen Transformation allein auf das Know-how und das Engagement der Mitarbeitenden zu vertrauen. Die Einbindung von externen Beratern haben wir explizit ausgeschlossen. Das Transformations-Team rekrutierte sich zunächst aus 35 Mitarbeitenden aus unterschiedlichen Bereichen und Hierarchiestufen. Das Team arbeitete ab Mitte Mai 2020 mit agilen Methoden (z. B. Scrum, Design-Thinking) in fünf verschiedenen Arbeitsgruppen (Abb. 2). Das Methoden Know-how wurde dem Transformations-Team in internen Schulungen vermittelt. KOMSA nutzte bereits viele Jahre agile Methoden und Lean Management-Konzepte in der IT und Logistik. Wir nutzten die eigenen Expert/innen als Trainer für das Transformations-Team. Die Inhalte der internen Schulung lehnten sich u. a. an die im Buch „Six Sigma + Lean Toolset“ (Meran et al. 2012) beschriebenen Werkzeuge an.

Bis zum Beginn der sächsischen Sommerferien (Ende Juli 2020) sollten die fünf Schwerpunktthemen detaillierter analysiert und ein Konzept erarbeitet werden. Der Abschluss der Umsetzungsmaßnahmen war für das Jahresende 2020 vorgesehen. Das Konzept sollte den Weg zu einer kompakteren Organisation und zu dynamischen Netzwerken aufzeigen sowie zur Reduktion der Kosten und der Kapitalbindung führen.

Die räumliche Organisation nahm das Projektteam unter Beachtung der Hygienevorschriften selbst in die Hand. Eine nicht mehr genutzte Lagerhalle in unmittelbarer Nähe zum Hauptsitz der KOMSA wurde kurzerhand zum „Innovation-Hub“ umgebaut. Die räumliche Trennung erschien sinnvoll, um sichtbar etwas anders zu machen als bisher. Es gab keine festen Arbeitsplätze und -zeiten sowie viel Raum für Kreativität. Die Wände wurden für die Abbildung von Ideen, Regeln, Prozessen und Ergebnissen genutzt. Auf einer Wäscheleine konnten alle Team-Mitglieder Karten aufhängen, auf denen sie ihre Empfindungen und Gedanken zur Transformation 2020 aufgeschrieben hatten. In der wöchentlichen Retrospektive (Element der Scrum-Methodik zur Verbesserung der Zusammenarbeit) wurden einzelne Karten herausgegriffen und in der Gruppe besprochen. Zudem schuf die IT die technischen Voraussetzungen, dass die Team-Mitglieder problemlos entweder im Innovation-Hub oder im HomeOffice am Projekt mitarbeiten konnten. Die wöchentliche Abnahme des Sprints (Prozess der Scrum-Methodik, indem bestimmte Aufgaben in einer vorgegebenen Zeit bearbeitet werden) fanden in Hybrid-Veranstaltungen statt.

## Wenn jeder an sein Kästchen denkt, denkt kaum einer an das Ganze

Die Vereinfachung der Konzernstruktur und Anpassung des Portfolios im Handels- und Dienstleistungsgeschäft waren wesentliche Ergebnisse der Transformation 2020. Aus 20 Konzerngesellschaften wurden 5 operative Bereiche. Durch die neue rechtliche Struktur halbierten sich die internen Leistungsverrechnungen und erhöhte sich die Transparenz.

Um die Arbeit im Tagesgeschäft effizienter zu bewältigen, bündelte KOMSA die fachlichen Kompetenzen der gesamten Gruppe in größeren funktionalen Einheiten. Die Methodik zur Verbesserung der Zusammenarbeit, wie sie z. B. im Buch „Tasks & Teams“ beschrieben sind (Große und Tillmanns-Estorf 2018; es wurde jedem Teammitglied zu Beginn des Projekts zur Verfügung gestellt), hat das Projektteam konsequent angewandt: Jeder Mitarbeiter wird disziplinarisch einem sog. „Core Team“ zugeordnet. Die Core Teams stellen das Fundament dar. In diesen Teams werden die Aufgaben bearbeitet, in denen das Ergebnis klar und der Prozess strukturiert ist. Aus den einheitlichen Bearbeitungsstandards innerhalb des Core Teams, der Reduktion überflüssiger Aufgaben und der Definition der wichtigen Schnittstellen ergaben sich die größten Produktivitätsgewinne. Zudem konnte KOMSA mit den größeren Einheiten den Wünschen nach Teilzeitbeschäftigung und Elternzeiten noch besser entsprechen.

**Figure 3:**
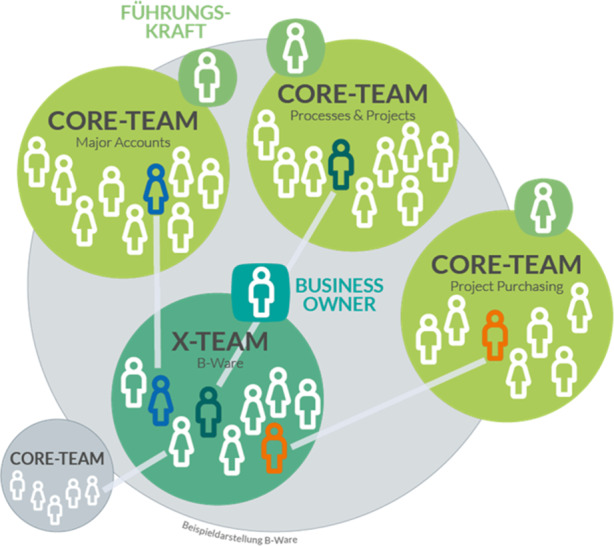

Die gravierendste Veränderung war die Einführung sog. „X-Teams“. Mit den X‑Teams schaffte KOMSA eine Struktur, neue Ideen ohne den Aufbau neuen Personals zu verwirklichen. Zudem gestalteten X‑Teams die Bearbeitung bereichsübergreifender Aufgaben und von temporären Projekten effizienter (Abb. 3). Die X‑Teams werden vom Vorstand bestimmt und bearbeiten ein vorgegebenes Thema.

Innerhalb des X‑Teams gibt es keine Hierarchie. Die Teammitglieder rekrutieren sich aus den Core-Teams und wählen aus ihrem Kreis einen „Business Owner“. Dieser ist Hauptansprechpartner des X‑Teams, hat Budgetverantwortung und entscheidet über die Abnahme von vorgestellten Arbeitsergebnissen. Er vereinbart mit den Führungskräften der Core-Teams das zeitliche Kontingent der Personalkapazität. Der zeitliche Einsatz kann zwischen 20 % und 100 % variieren. Die Vertretungs- und Urlaubsregelungen des Core-Teams gelten auch für die X‑Teams.

Die X‑Teammitglieder vereinbaren gemeinsam nicht nur die Prinzipien und Instrumente der Zusammenarbeit, sondern auch die Verantwortlichkeiten und Aufgaben. Die Aufgaben sind unabhängig von Personen. Jedes Teammitglied kann frei entscheiden, bei welchen Aufgaben es seine Fähigkeiten einbringt. Durch die freie Aufgabenwahl entsteht eine hohe Identifikation und Umsetzungsgeschwindigkeit. Gleichzeitig entsteht für jedes einzelne Teammitglied die Verantwortung, die versprochene Aufgabe ganzheitlich und termingerecht zu bearbeiten. Alle Mitglieder des X‑Teams vertrauen aufeinander, entscheiden selbst, wie sie die Aufgabe bearbeiten, und achten darauf, dass das gemeinsam gesetzte Ziel erreicht wird.

Neben der Organisation ist auch die Verzahnung der für das Geschäftsmodell relevanten Kennzahlen mit der Steuerung der Geschäftsprozesse für KOMSA erfolgskritisch. In der Vergangenheit wurden die wichtigen Geschäftsentscheidungen hauptsächlich auf Basis des Deckungsbeitrags getroffen. Um das Netto-Umlaufvermögen systematisch zu senken und die Fixkosten besser zu kontrollieren, reichte diese Kennzahl nicht aus. Deshalb führte KOMSA mit der Transformation 2020 den Return on Capital Employed (ROCE) als neues führendes Steuerungsinstrument ein. Die Kennzahl schlägt die Brücke zwischen operativem Ertrag und Kapitalbindung und fand ebenfalls Eingang in das variable Vergütungssystem. Jeder Mitarbeiter ist seit dem Geschäftsjahr 2021/22 am Erfolg beteiligt und hat eine variable Vergütungskomponente, die sich am ROCE orientiert.

## Kognitive persönliche Weiterentwicklung in kleinen Gruppen

Die Einführung einer selbstorganisierten und agilen Form der Zusammenarbeit erforderte ein neues Führungsverständnis. Um unsere neuen Vorstellungen über die Art und Weise der Zusammenarbeit mit denen der Führungskräfte abzugleichen, schrieben wir als Vorstand alle Führungsstellen intern aus. Diese Vorgehensweise war deshalb bemerkenswert, weil viele Kandidaten bereits über langjährige Führungserfahrung im Unternehmen verfügten. Zum anderen sah die neue Struktur nur noch rund halb so viele Führungsstellen vor.

Für die Auswahl der Führungskräfte konzipierte die Abteilung People & Culture ein Auswahlverfahren. Es sah u. a. vor, dass die Kandidaten auf Basis eines 100-Tage-Plans erläuterten, wie sie ein Umfeld für eigenverantwortliches Arbeiten schaffen, Erwartungen ausdrücken sowie Orientierung und Feedback geben. Am Auswahlverfahren konnten alle bestehenden Führungskräfte und Mitarbeitende, die in der neuen Struktur eine Führungsrolle übernehmen wollten, teilnehmen. Rund die Hälfte der Bewerber setzte sich im Auswahlverfahren nicht durch. Jeder abgelehnte Bewerber erhielt eine wertschätzende Rückmeldung zu seiner Leistung im Auswahlverfahren sowie ein Angebot für eine fachgerechte Aufgabe ohne Führungsfunktion in einem Core-Team. Einige abgelehnte Bewerber verließen KOMSA in den Folgemonaten. Die Kandidaten, die sich im Auswahlverfahren durchsetzten, erhielten ebenfalls ein umfassendes Feedback zum vorgestellten 100-Tage Programm und wurden zur KOMSA Leadership Journey eingeladen. Das Führungskräfteentwicklungsprogramm teilte sich in drei Module mit je 2 Tagen auf.

**Figure 4:**
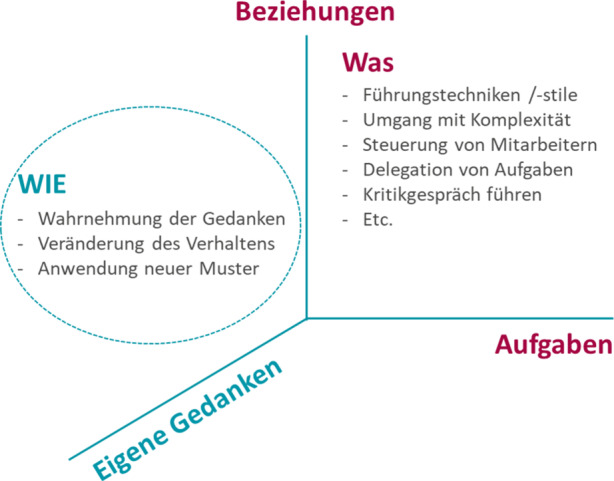

Viele der ausgewählten Führungskräfte erwarteten von der KOMSA Leadership Journey ein „Handbuch“ für die Führung. Im Vorstand waren wir davon überzeugt, dass der „Werkzeugkoffer für die Führung“ nur unzureichend berücksichtigt, dass jede Führungskraft sich in unterschiedlichen sozialen Kontexten bewegt und jeder Mitarbeiter sehr unterschiedlich zu führen ist. Als Vorstand vertraten wir die Auffassung, dass die Wirksamkeit einer Führungskraft vom dem „Wie“ der individuellen Ausgestaltung der Rolle abhängig ist (Abb. 4). Das Verhalten wird maßgeblich von den eigenen mentalen Modellen bestimmt (vgl. Haidt 2006). Die KOMSA Leadership Journey setzte deshalb bei den Führungskräften an – es ging um die Reflexion der eigenen Führungsrolle sowie die Formulierung von Veränderungswünschen.

**Figure 5:**
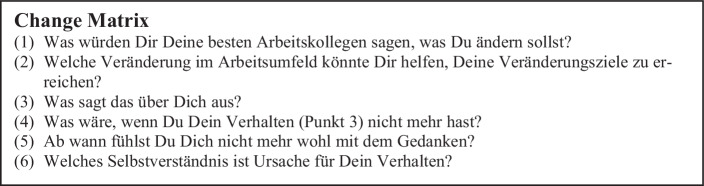

KOMSA teilte die über 50 Führungskräfte in „Home Groups“ auf. Jede Home Group setzte sich aus maximal sechs Führungskräften aus unterschiedlichen Bereichen, Hierarchiestufen und Geschlecht zusammen. Die Home Groups sollten für das „kollegiale Coaching“ auch nach der KOMSA Leadership Journey fortbestehen. Zum überwiegenden Teil arbeiteten die Mitglieder innerhalb einer Home Group zuvor nicht zusammen, sondern kannten sich nur vom Sehen. Mit Hilfe von strukturierten Fragen sollte jede Führungskraft für sich eine „Change Matrix“ erarbeiten (Abb. 5). Die Erstellung begann bereits im ersten Modul und die Gedanken konkretisierten sich im weiteren Verlauf des Programms. Ein externer Moderator unterstützte die Führungskräfte im ersten Modul bei der Erarbeitung der Change Matrix.

Bei der Konzeption der KOMSA Leadership Journey fragten wir uns im Vorstand, ob die Führungskräfte in den Home Groups eine offene Diskussion führen und die Problempunkte auch tatsächlich ansprechen würden. Die Diskussion eines Fremdbildes war nicht möglich, weil diese nicht systematisch erhoben waren. Die Situation war zusätzlich erschwert, weil alle Workshops aufgrund der Covid-19 Pandemie virtuell stattfinden mussten. Es stellte sich dann aber heraus, dass für die Führungskräfte die Home Groups der wichtigste Baustein der Leadership Journey war. Sie fassten Vertrauen zueinander. Das konstruktive Feedback in den kleinen Gruppen empfanden die Führungskräfte hilfreich. Durch die gute Arbeitsatmosphäre in der Home Group war es möglich, kognitive Verzerrungen zu erkennen, eine Bereitschaft für die Anpassung des eigenen Verhaltens zu entwickeln und Aufgaben zu priorisieren.

Die KOMSA Leadership Journey bestand aus mehreren inhaltlichen Bausteinen. Wir wollten den neuen Führungskräften neben der Reflexion des eigenen Verhaltens auch das Rüstzeug für das Ausfüllen der neuen Aufgabe geben. So erklärte wir als Vorstand zu Beginn das neue KOMSA Führungsverständnis. Im Anschluss diskutierten die Führungskräfte Theorien der Entscheidungsfindung und Methoden zur Verbesserung der eigenen Resilienz. In weiteren Bausteinen wurden die Führungskräfte in Gruppenarbeiten mit Fallbeispielen aus dem Führungsalltag sowie mit fachlichen Themen aus den Bereichen Finanzen, Recht und Compliance ausgebildet.

## KOMSA hat das Unmögliche möglich gemacht

KOMSA schloss das Projekt im Dezember 2020 erfolgreich ab. Mit der Transformation 2020 vereinfachte KOMSA die rechtliche Struktur und etablierte eine moderne Führungs- und Steuerungsstruktur. Die Führungskräfte fanden durch die Leadership Journey zu einer Einheit zurück. Die Home Groups sind heute fester Bestandteil der Organisation und werden von den Führungskräften selbstständig als Instrument des kollegialen Coachings genutzt. Durch die klare Abgrenzung von Distribution und Dienstleistungen kann KOMSA besser auf die unterschiedlichen Markt- und Wettbewerbsstrukturen eingehen. Rund zwei Drittel der Marken wurden nach dem Projekt nicht mehr fortgeführt und aufgegeben. Die Positionierung und die Kraftfelder der verbliebenen Marken wurden geschärft. Möglich war die Veränderung durch die Offenheit, Neugier und Entschlossenheit der Mitarbeitenden, neue Wege zu gehen. Alle Beteiligten ließen sich mit dem Projekt Transformation 2020 auf eine neue Art der Projektführung ein. Die aus der Scrum-Methodik abgeleiteten Prinzipien und Verhaltensregeln setzten während des Projekts den Pioniergeist frei und unterstützten die hohe Umsetzungsgeschwindigkeit.

Die Veränderungen spiegelten sich auch in der finanziellen Entwicklung wider. Im Geschäftsjahr 2020/21 fand KOMSA wieder zu Wachstum zurück. Die Gesamtleistung stieg um über 15 % auf 1,4 Mrd. € – ein Rekordwert in der fast 30-jährigen Unternehmensgeschichte. Das operative Ergebnis (EBITDA) wuchs um 20 %, und der frei verfügbare Cashflow verdreifachte sich. Das für KOMSA wichtige Rating verbesserte sich auf Investment Grade.

Trotz aller Erfolge hat sich die Arbeitslast in vielen Bereichen in den ersten sechs Monaten des Geschäftsjahrs 2021/22 noch nicht spürbar reduziert. Es bleibt für uns alle weiterhin schwierig, unser eigenes Verhalten und das von anderen zu ändern. Unsere Verhaltensmuster hängen wie bisher von unbewussten Faktoren und wahrgenommenen Erwartungen der Umwelt ab. Alle Führungskräfte beschlossen deshalb, die Leadership Journey fortzusetzen. Jeder war motiviert, die Dinge zu ändern und zu einem wirkungsvolleren Führungsverhalten zu finden. So stellen Führungskräfte während der regelmäßig stattfindenden Treffen z. B. ihre Erfahrungen im Zusammenhang mit der Verhaltensveränderung vor. Dabei diskutieren wir in der Gruppe offen Erfolge und Herausforderungen.

Mit dem neuen 360 Grad KOMSA Collaboration Navi wird jede Führungskraft ab 2022 überprüfen können, ob sie die Selbststeuerung statt Kontrolle auch tatsächlich umsetzt und wie sie von ihrem Umfeld wahrgenommen wird.

Auch die Zusammenarbeit von Core-Teams und X‑Teams wird von uns regelmäßig überprüft. Wir haben in den ersten sechs Monaten festgestellt, dass die neuen Rollen noch nicht immer ausgefüllt werden. Für die Führungskräfte der Core-Teams ist es zum Teil noch sehr gewöhnungsbedürftig, dass ein Teil ihres Teams in einem selbstorganisierten X‑Team arbeitet. Auch für die Teammitglieder eines X‑Teams sind die neue Arbeitsweise und die Trennung der Rollen ungewohnt. Bis die neuen Rollen reibungslos umgesetzt werden, wird es noch einige Zeit brauchen – das Fundament ist jedoch mit der Transformation 2020 gelegt.

Das Interesse der Mitarbeitenden an den agilen Arbeitsmethoden ist in der gesamten Unternehmensgruppe groß. Das ist gut so, denn die Notwendigkeit der Veränderung endet nicht. Wir haben im Vorstand deshalb beschlossen, die Ausbildung in agilen Methoden und Lean Management Konzepten weiter zu forcieren. Mit der gewonnenen Erfahrung aus dem Projekt Transformation 2020, dem Methodenwissen und der breiten Akzeptanz der neuen Arbeitsweisen ist KOMSA gut aufgestellt, um den sich abzeichnenden Veränderungen zu begegnen.

## Persönliche Sicht der KOMSA Aufsichtsratsvorsitzenden Kerstin Grosse

Familienunternehmer der ersten Generation gründen, entwickeln, kämpfen, riskieren alles, was sie besitzen, überleben, managen Wachstum und Krisen – und stehen auf einmal vor einer bisher nicht gekannten Herausforderung: ihr Baby in fremde Hände zu geben, wo es doch keine sichereren, behütenderen und besseren Hände als die eigenen gibt.

Jedes Familienunternehmen hat eine einzigartige Geschichte und funktioniert auf seine eigene, individuelle Weise. Diese Faktoren legen dem ersten Management, das den Gründern nachfolgt, besondere Herausforderungen auf. Es braucht einen führungsstarken, erfahrenen und kundigen Nachfolger. Diese Person muss auch in bewahrender und zugleich entwickelnder Weise zur Unternehmenskultur und -mentalität passen.

Im besten Falle initiieren Gründer diesen Prozess selbst, im ungünstigen Fall trifft ein Führungswechsel das Unternehmen, ggf. den Unternehmer und dessen Familie völlig unvorbereitet aus heiterem Himmel.

Mein Mann, ein gebürtiger Schwede, gründete KOMSA 1992 im Alter von 53 Jahren und entwickelte es zum heute größten Familienunternehmen Ostdeutschlands – mit Mitarbeitern, die vor allem in der ersten Hälfte der Unternehmensgeschichte mehrheitlich von der DDR-Planwirtschaft geprägt waren. Die ostdeutsch-schwedische Mischung und eine rasante Geschäftsentwicklung schufen in den 90er und 00er-Jahren eine sehr besondere Atmosphäre des erfolgreichen Aufbruchs. KOMSA war bekannt für ein innovatives und verantwortungsförderndes Milieu. Mit dem weiteren Wachstum entstand jedoch parallel eine komplexe Konzernstruktur mit überbordender Administration und „Beschäftigung mit uns selbst“, was unsere einstige Flexibilität und Schnelligkeit am Markt zunehmend lähmte und sich letztlich in den Geschäftsergebnissen niederschlug. Obwohl wir uns dieser Situation sehr bewusst waren, waren wir nicht in der Lage, eine umfassende Reorganisation aus unternehmenseigenen Kräften anzugehen.

Vor diesem Hintergrund erfolgte im Frühjahr 2020 erstmals die Berufung eines unternehmensfremden Managers als CEO/CFO mit dem Ziel, das Unternehmen nach der Ära der Geschäftsführung durch die Eigentümer und dem nachfolgenden ersten Vorstand aus langjährigen KOMSA-Mitarbeitern weiterzuentwickeln, strategisch neu aufzustellen und gleichzeitig durch einen umfassenden und tiefgreifenden Transformations- und Optimierungsprozess die Unternehmensgruppe wieder zukunftssicher und stabil zu machen.

In der ersten gemeinsamen Sitzung des Aufsichtsrates mit dem designierten CEO wurden sehr offen die gegenseitigen Erwartungen an die Ausgestaltung der künftigen Zusammenarbeit besprochen. Zum Beispiel die Häufigkeit und Tiefe der Berichterstattung des Vorstandes an den Aufsichtsrat, aber auch Wünsche bezüglich der darüber hinausgehenden gemeinsamen informellen Kommunikation. Zu unserer Überraschung und Freude war der Wunsch nach regelmäßigem Austausch beim neuen CEO deutlich größer als erwartet. Die Kommunikation mit dem bisherigen Vorstand war geprägt durch Zurückhaltung und Formalität, ein Ideenaustausch bzw. frühzeitige Signale bezüglich aufkommender Schwierigkeiten, wie es sich der Aufsichtsrat gewünscht hätte, fand eher nicht statt.

Ein so offensives Kommunikations- und Informationsverhalten seitens des CEO trug von Beginn an entscheidend zur Bildung einer starken Vertrauensbasis zwischen CEO/Vorstand und Aufsichtsrat bei, welche wiederum unabdingbar dafür ist, dass die langjährigen Gründungsvorstände und Eigentümer den nötigen Abstand entwickeln können, den ein Fremdmanagement braucht.

Dieses Loslassen ist ein abrupter Rollenwechsel, der von den Eigentümern viel Disziplin erfordert und glasklare Regeln benötigt. So wurde z. B. die Beschäftigung mit detaillierten Sachverhalten und Fragen auf operativer Ebene zum absoluten „No-Go“. Strikte Regeln galten ab sofort auch für die Kommunikationslinien. Was ehemals ein riesiger Pluspunkt in der Unternehmenskultur war und etwas Typisches für eigentümergeführte Unternehmen ist, war von Stund an tabu: nämlich, dass die (jetzt ehemaligen) Chefs durch die Hallen gehen und mit Mitarbeitenden sprechen. Das fällt schwer!

Eine neue Führung braucht neben komplett freier Hand in ihren Entscheidungen vor allem jedoch die 100 %ige Rückendeckung durch die bisherigen Leitfiguren, die seit Unternehmensgründung für die Mitarbeitenden die zentralen Personen des Unternehmens waren und denen in der Regel noch immer große Wertschätzung und Respekt und mitunter auch nostalgische Sehnsucht entgegengebracht werden. Der Aufsichtsrat musste und wollte sein vollumfängliches Vertrauen dem neuen Führungsteam gegenüber in die Organisation ausstrahlen, was durch die Covid-Einschränkungen stark erschwert wurde. Aber selbst durch einen Like oder Kommentar in der KOMSA-App kann man entsprechende Signale senden.

Sehr schnell wurde der von uns erwartete Vorteil des „externen Blicks“ tatsächlich deutlich: Pierre-Pascal Urbon analysierte und bewertete die vorhandenen Strukturen ohne (störende) Relation zur Geschichte, ohne die damit verbundenen Emotionen – sondern unvoreingenommen und objektiv. In seiner Analyse wurde er nicht beeinflusst von lange zurückliegenden und irgendwann „Gesetz gewordenen“ Beschlüssen der Gründungsvorstände, er verteidigte nicht unbewusst das „historisch Gewachsene“, vielmehr hinterfragte er alles Vorhandene und bewertete es nach dessen Wert für die zukünftige Entwicklung des Unternehmens.

Der Aufsichtsrat schloss sich nahezu vollständig seiner Analyse an. Nur ganz wenige Punkte wurden diskutiert, und ein Konsens wurde erarbeitet. Ebenso folgten wir der Wahl der daraus resultierenden Maßnahmen des Vorstandes – auch wenn diese Unmut oder Widerstände in der Organisation erwarten ließen, was in einem so umfangreichen Transformationsprozess nicht ausbleibt.

Wir waren vorbereitet, dass sich eventuell langjährige Mitarbeiter an uns wenden würden, um Sorgen zu teilen oder sich über Entscheidungen oder Akteure zu beschweren. Hier stand der Aufsichtsrat stets 100%ig hinter dem Vorstand. Denn mit dem kleinsten falschen Signal, und sei es nur ein Augenrollen, hätten wir dessen Autorität geschwächt und den gesamten Prozess empfindlich gestört.

Pierre-Pascal Urbon befähigte die Organisation, aus sich selbst heraus die Transformations-Ideen zu erarbeiten und umzusetzen – aus unserer Sicht ein Meilenstein in der Führung. Die von ihm vorgeschlagenen neuen Arbeitsweisen (Scrum, Design Thinking) wurden von der Organisation gierig aufgesogen und angewandt. Es stellte sich sehr schnell die Aufbruchstimmung aus den 90ern wieder ein, eine energiegeladene, kreative Atmosphäre durchzog das Unternehmen. „T2020“ stellte das Kästchen-Denken in Frage. Genauso hatten wir jahrelang bis weit in die 2000er gearbeitet, ohne limitierendes Organigramm.

Die Erwartungen an die Ergebnisse waren auch bei den im Projekt nicht direkt beteiligten Mitarbeitenden groß. Dieses Klima in der Organisation, gekoppelt an eine engmaschige transparente Kommunikation des Vorstandes uns gegenüber bestärkte unser Vertrauen in das Führungsteam.

Die straffe und zugleich anspornende und treibende Führung, konsequente Verantwortungszuordnung, Transparenz und nicht zuletzt die erfreuliche Verbesserung unserer betriebswirtschaftlichen Kennzahlen und der Gesamtleistung des Unternehmens erzeugten beim Aufsichtsrat das Gefühl „Sie haben es im Griff.“

Die große Herausforderung besteht aus meiner Sicht jetzt darin, den Magnetismus „zurück zum Alten“ zu unterbinden, den Klebstoff aus der Organisation zu entfernen, der die Beteiligten, insbesondere langjährige Mitarbeiter, am Bekannten und Liebgewordenen festkleben lässt.

Mit der Organisation ohne Organigramm hat der Vorstand die Vorteile eines Familienunternehmens genutzt, die Tugenden des einstmals kleinen „Underdogs“ KOMSA aus den 90er-Jahren reaktiviert und den Begriff der Veränderung wieder positiv konnotiert. Gekoppelt an das Mindset des Loslassens der ehemaligen Vorstände hat das Unternehmen in weniger als einem Jahr einen beachtlichen Entwicklungssprung vollzogen.

## Persönliche Sicht von Andrea Fiedler-Braunschweig, KOMSA Vice President Corporate Communication

„Jede Führungskraft und jeder Mitarbeiter kann sein Interesse für unsere neue Führungsrolle bekunden.“ Mit diesen Worten hatte unser Vorstand den Startschuss für eine komplette Reorganisation der Führungsmannschaft gegeben. Hatte ich richtig gehört? Ich soll mich auf meinen Job, den ich seit Jahren mache, neu bewerben? Was sich zunächst wie ein Aprilscherz anfühlte, erwies sich als sehr lehrreiches Führungskräfte-Entwicklungsprogramm.

Wenn sich ein Unternehmen derart verändert und entwickelt, erfordert das ein neues Führungsverhalten. Andere Unternehmen hätten hierfür die Führungsmannschaft ausgetauscht. Nicht so KOMSA. Der Vorstand wollte, dass sich die Veränderung aus eigener Kraft generiert. Bemerkenswert! Das Ziel war dabei klar formuliert: Raus aus dem Tagesgeschäft, in dem so viele von uns steckten. Rein in eine neue Führungsrolle, die es sich zur Aufgabe macht, ein Team zum Erfolg zu führen, statt selbst der beste Fachexperte im Team zu sein. Eine Führungsrolle, die Mitarbeitern Orientierung gibt, die priorisiert und das Team sowohl fordert als auch fördert.

Einige Gesprächsrunden und Präsentationen später stand die künftige Führungsmannschaft fest. Und dahinter eine komplett neue Organisationsstruktur mit deutlich weniger Führungskräften und dafür größeren Führungsspannen, die es gar nicht mehr zulassen, zu tief in die fachlichen Themen einzusteigen.

Was nun vor uns lag, war eine gemeinsame Lernreise. Was ist unser Verständnis von Führung? Nach welchen Werten und Regeln wollen wir unsere Teams führen und entwickeln? Wie erhalten wir trotz aller Veränderungen unsere Unternehmenskultur, in der es einfach Spaß macht, zu arbeiten? Und wie schaffen wir es, dass wir trotz unserer breiten Aufstellung über alle Teams hinweg an einem Strang ziehen?

Über Vorträge und Workshops erarbeiteten wir unseren Fahrplan. Das Organisationsteam war überzeugt davon, dass wir das aus eigener Kraft schaffen, und setzte auf kleine Gruppen aus 4 bis 6 Führungskräften. Unsere „Home Groups“. Bunt durchmischt über alle Fachbereiche, Altersgruppen und Erfahrungslevel hinweg. Und so fanden wir uns plötzlich mit Führungskollegen in einer Gruppe wieder, mit denen es bislang kaum berufliche Berührungspunkte gab. Und wir stellten fest, wie viele Parallelen unsere Arbeit hat und wie sehr wir uns untereinander helfen können. Insofern verwundert es nicht, dass diese Gruppen nach wie vor existieren. Die Home Groups sind ein Ort des Vertrauens und der Reflexion für uns geworden – der Name ist also Programm. Wir treffen uns regelmäßig, tauschen uns zu den Führungsfragen im Alltag aus und stützen uns gegenseitig, wenn wir vor neuen Aufgaben stehen.

Ist heute alles besser? Nein. Noch (!) nicht. Denn Verhaltensmuster ändern sich nicht von heute auf morgen. Eingefahrene Prozesse ändern sich nicht von jetzt auf gleich. Und es braucht eben seine Zeit, bis sich neu formierte Teams aufeinander einstellen. Aber wir haben uns auf den Weg gemacht und packen Stück für Stück die Dinge an, die uns heute noch ausbremsen.

## Footnotes

1 Der Team-Coach ist bei KOMSA Ansprechpartner für berufliche und private Belange der Mitarbeiter mit Auswirkungen auf das Arbeitsleben. Der Team-Coach nimmt sich Zeit, hört zu, informiert und unterstützt in Konfliktsituationen.
